# Enhanced supportive care for advanced cancer patients: study protocol for a randomized controlled trial

**DOI:** 10.1186/s12912-022-01097-5

**Published:** 2022-12-02

**Authors:** Yun Young Choi, Sun Young Rha, Sungkun Cho, Hye Sun Lee, Bomi Hong, Jiyeon Lee

**Affiliations:** 1 College of Nursing and Brain Korea 21 FOUR Project, Yonsei University, Seoul, Korea; 2 College of Medicine and Yonsei Cancer Center, Yonsei University, Seoul, Korea; 3 Department of Psychology, Chungnam National University, Daejeon, Korea; 4 College of Medicine, Biostatistics Collaboration Unit, Yonsei University, Seoul, Korea; 5 College of Nursing and Mo-Im Kim Research Institute, Yonsei University, 50-1 Yonsei-ro, Seodaemun-gu, Seoul, Korea

**Keywords:** Symptom, Coping, Quality of life, Self-efficacy, Nursing intervention, Advanced cancer

## Abstract

**Background:**

Early palliative care along with standard cancer treatments is recommended in current clinical guidelines to improve the quality of life and survival of cancer patients. This study protocol aims to evaluate the effect of “Enhanced Supportive Care”, an early primary palliative care provided by nurses.

**Methods:**

A randomized controlled trial (RCT) will be conducted including advanced cancer patients scheduled for first-line palliative chemotherapy (*N*=360) and their caregivers in South Korea. Participants will be randomly assigned to the intervention or control group in a 1:1 ratio. Participants in the intervention group will receive the “Enhanced Supportive Care”, which provides five sessions of symptom management and coping enhancement counseling by nurses. The control group will receive symptom monitoring five times. The primary endpoints are symptoms, coping, and quality of life (QoL) at 3 months. Secondary endpoints are symptoms, coping, and QoL at 6 months, depression and self-efficacy for coping with cancer at 3 and 6 months, symptom and depression change from baseline to 3 months, survival at 6 and 12 months among patients, and depression among caregivers at 3 and 6 months.

**Discussion:**

This RCT will evaluate the effects of “Enhanced Supportive Care” on symptoms, depression, coping, self-efficacy for coping with cancer, QoL and survival of patients, as well as depression of caregivers. It will provide evidence of a strategy to implement early primary palliative care provided by nurses, which may consequently improve cancer care for newly diagnosed patients with advanced stage cancer.

**Trial registration:**

ClinicalTrials.gov, NCT04407013. Registered on May 29, 2020, https://www.clinicaltrials.gov/ct2/show/study/NCT04407013. The protocol version is ESC 1.0.

## Background

The ultimate goal of care for advanced cancer patients is to improve quality of life (QoL). Supportive care refers to care aimed at improving the QoL of patients experiencing life-threatening diseases. Patients with advanced cancer suffer from various symptoms such as pain, fatigue, and depression, which are known to negatively affect the patient's quality of life [1–5].

The WHO defines palliative care as early recognition of pain, physical, psychosocial, and spiritual problems faced by patients and their families related to life-threatening diseases and improving the quality of life through faithful assessment and intervention [6].

Palliative care is not limited to the end-of-life phase. Early palliative care provided within 8 to 12 weeks of advanced cancer diagnosis demonstrated its effectiveness in managing depression and improving quality of life and survival [7–13]. Studies have reported that early palliative care moderates the influence of depression on survival [14–16]. Survival after receiving early palliative care is reported as 4.56 months, which is comparable to 3.43 months of survival from recently licensed new cancer medicines [10, 17]. Although providing supportive care would not reduce the actual burden on family caregivers [18, 19], it is known to ameliorate symptoms of depression experienced by family caregivers [20]. Clinical guidelines recommend providing early palliative care within 8 weeks of cancer diagnosis along with standard cancer treatment for advanced cancer patients [21–23].

The provision of early palliative care could be achieved by integrating primary palliative care into standard cancer care. Primary palliative care denotes basic features of palliative care such as basic symptom management to be delivered by any clinicians caring for advanced cancer patients [24, 25]. Clinical practice guidelines for quality palliative care suggest essential palliative care skills are needed by all clinicians [23]. To provide primary palliative care, any clinician, not just a palliative care expert, needs to play an active role in palliative care provision through education and training [26, 27]. Providing primary palliative care as a part of standard care will meet patients’ supportive care needs and identify those at need for specialist palliative care.

Difficulties in accepting the term ‘palliative care’ at times of advanced cancer diagnosis have been an issue. The term ‘Enhanced Supportive Care’ has been suggested by NHS England to differentiate it from supportive care, which means the care of patients receiving active treatment [28] and palliative care at the end of life [29]. “Enhanced supportive care” in the current study refers to ‘early primary palliative care’ provided by nurses at the staff level.

Symptom management is a major element of palliative care [27, 30]. Symptom management is suggested to promote coping with cancer among patients [31]. According to the study by Bischoff et al. [32], which analyzed topics of actual palliative care encounters, symptom management was the most commonly addressed issue (92% of initial care and 93% of follow-up care). Digital symptom monitoring during routine clinical encounters for palliative chemotherapy resulted in less decline in health-related quality of life, less frequent emergency room visits or hospitalizations, and extension of survival [33]. The second most frequently applied palliative care component was coping interventions, which were identified in 64.2% of encounters for palliative care [34]. Cancer patients who demonstrated negative coping experienced a higher level of depression, a significantly higher expression of major depressive episodes at 1 year after diagnosis [35], and a lower quality of life [36].

Nurses have played an important role in the interprofessional collaborations that provide palliative care [21, 22, 30]. The current study examined the effects of “Enhanced Supportive Care”, which was developed based on previous studies, a literature review, and expert opinions on symptom management and coping enhancement provided by staff-level nurses as primary palliative care. The “Enhanced Supportive Care”, comprising symptom management and coping enhancement counseling, will be provided during outpatient visits beginning with the time of advanced cancer diagnosis and during initial palliative chemotherapy cycles. Symptom management and monitoring will be provided according to evidence-based symptom management education. Coping enhancement counseling based on the principles of self-efficacy for coping with advanced cancer [37] and Acceptance and Commitment Therapy (ACT) [38] will be provided considering the role of coping on QoL [39, 40]. The effect of the “Enhanced Supportive Care” as early primary palliative care provided by nurses along with standard cancer treatment will be evaluated.

## Methods

### Aim

The aim of this randomized controlled trial was to compare “Enhanced Supportive Care” to symptom monitoring in terms of effects on advanced cancer patients’ symptoms, depression, coping, self-efficacy for coping with cancer, quality of life, survival, and depression in caregivers.

### Design

**Fig. 1:**
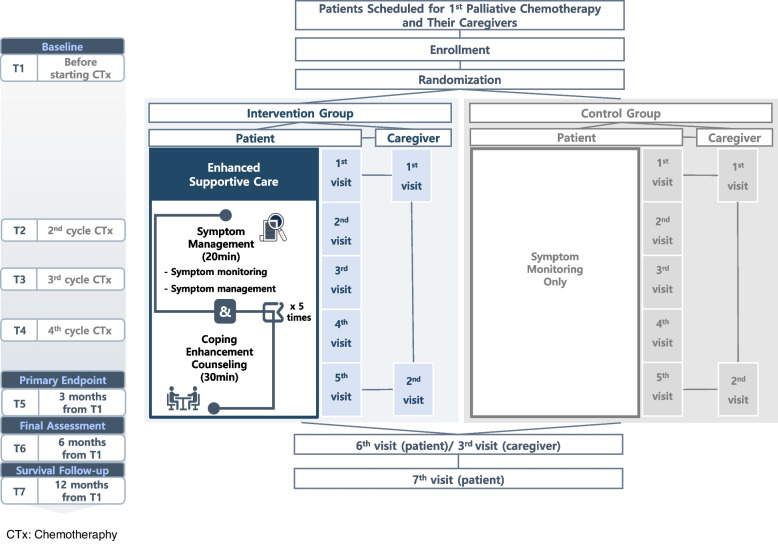
Study design

A randomized controlled trial will be conducted by randomly allocating participants in a 1:1 ratio to the intervention or control arm. Participants in the intervention arm will receive the “Enhanced Supportive Care” consisting of symptom management and five sessions of coping enhancement counseling, while control group participants will receive symptom monitoring only. This protocol followed the SPIRIT (Standard protocol items: recommendations for interventional trials) guidelines for reporting protocols [41] (Fig. 1).

### Setting

Patients and their caregivers will be recruited from a single cancer center that belongs to a tertiary hospital located in Seoul, Korea.

### Participants

#### Inclusion criteria

- **Patient:**
- Adults (age ≥ 19)
- Diagnosis of advanced cancer
- Scheduled to receive first-line palliative chemotherapy
- European Cooperative Oncology Group Performance Status ≤ 2
- **Caregiver:**
- Adults (age ≥ 19)
- Family caregivers of cancer patients who agree to participate in the study

#### Exclusion criteria

Those diagnosed with cognitive or psychiatric problems and receiving treatment

### Randomization and blinding

The random allocation sequence was developed by the principal investigator (PI) before initiating patient enrollment. Research nurses will enroll and allocate patients according to the predefined sequence. Stratified permuted block randomization will be used to assign participants in a 1:1 ratio to the intervention or control group. Strata include the type of cancer (lung, breast, colorectal, gastric, liver/biliary/pancreas, or other cancers), age and sex of cancer patients. The minimization method will be applied after enrolling 60% of the participants in each cancer group to balance group allocation. Blinding is not considered applicable based on the type of intervention provided.

### Intervention

The intervention will be “Enhanced Supportive Care”, consisting of 1) symptom management (symptom monitoring and management) and 2) coping enhancement counseling. The “Enhanced Supportive Care” sessions will be provided by nurses five times over three months. Administration of the sessions will take approximately 50 min per visit: 1) symptom management (20 min) + 2) coping enhancement counseling (30 min).

Fidelity of the intervention: Web-based symptom assessment and education application, symptom management education booklet for home use, and coping enhancement counseling protocols will strengthen the fidelity of the intervention.

Interventionist training and tracking: Before starting the intervention, a symptom expert (PI) will train research nurses in how to monitor symptoms and provide evidence-based symptom management education. A certified clinical psychologist will teach the principals of counseling and the third-wave psychotherapy Acceptance and Commitment Therapy (ACT) and train research nurses in coping enhancement counseling. Periodic retraining sessions will be held with a certified clinical psychologist.

### Web-based symptom management system

**Fig. 2:**
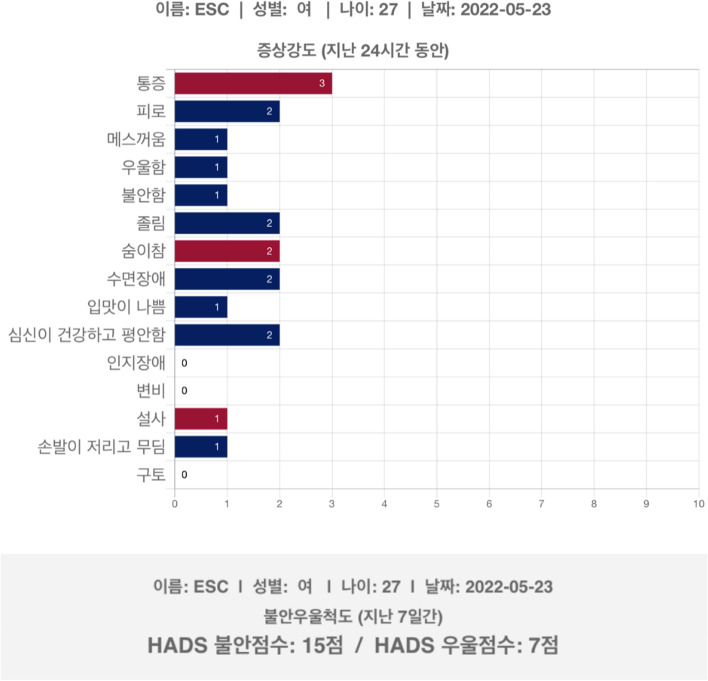
Example of symptom and depression assessment results from the “Cancer Symptom Management System” application

The “Cancer Symptom Management System” application for the iPad [42] was upgraded to a web-based application to improve access from multiple digital devices. The upgraded web-based "Cancer Symptom Management System" application was developed using Python 3.7 and the Django framework. Every chart was rendered using Chart.js (https://www.chartjs.org), a popular open-source JavaScript library for visualizing data. The Cancer Symptom Management system was designed so that users (researchers) had to log into access data. Patients’ symptoms will be monitored using the “Cancer Symptom Management System” on an iPad. The results from symptom monitoring will be presented as a colored bar graph to the patients’ oncologists. Anxiety and depression scores will be generated and presented along with the symptom bar graph (Fig. 2). The generated symptom bar graph and the anxiety and depression scores will be uploaded to the patients’ Electronic Medical Records (EMRs) for oncologists’ review. Symptom management education will be provided by research nurses based on the results of symptom monitoring reflecting patients’ needs, as per a previous study [42].

### Coping enhancement counseling based on principals of social cognitive theory and ACT

Protocols for coping enhancement counseling were developed based on the principles of social cognitive theory [43] and ACT [38]. Social cognitive theory will be applied to promote patients’ self-efficacy for coping with cancer. Patients’ current coping strategies in dealing with issues facing advanced cancer treatment and their effectiveness will be identified. As a strategy for mastery enhancement, patient-identified effective coping strategies will be promoted for use as resources for coping with cancer.

The principles of the ACT will be applied to help patients accept current emotions and thoughts experienced with advanced cancer diagnosis while finding and seeking life values. Committed actions toward life values will be encouraged and practiced. Coping enhancement counseling will be patient-centered because the program will help patients understand and accept their emotions, thoughts, and life values and choose their own committed actions. Throughout the program, participants will be encouraged to understand themselves as subjects of life (Table 1).

**Table 1:** Coping enhancement counseling for enhanced supportive care

Session	Theme	Contents
1	Introduction	∙ Introduction about the coping enhancement counseling ∙ Share patient experience: Change after cancer diagnosis and major emotions experienced
2	Current coping strategies in dealing with major emotions	∙ Check for changes in major emotions and reasons for change ∙ Current coping strategies in dealing with major emotions and its effectiveness ∙ Metaphor: Water jar with sand ∙ Mindfulness exercise ∙ Homework
3	Finding life values	∙ Check for changes in major emotions and reasons for change ∙ Current coping strategies in dealing with major emotions and its effectiveness ∙ Finding life values ∙ Mindfulness exercise ∙ Homework
4	Commitment toward life values	∙ Check for changes in major emotions and reasons for change ∙ Current coping strategies in dealing with major emotions and its effectiveness ∙ Commitment toward life values ∙ Metaphor: Passengers on the bus ∙ Metaphor: Chinese finger trap ∙ Homework
5	Moving out from mind and going back to life	∙ Check for changes in major emotions and reasons for change ∙ Current coping strategies in dealing with major emotions and its effectiveness ∙ Review of previous sessions ∙ Identify differences before and after the coping enhancement counseling participation ∙ Utilize currently working coping strategies ∙ Accept emotions and thoughts and committing toward life values ∙ Mindful exercise ∙ Farewell

### Study procedures

The intervention group will receive the “Enhanced Supportive Care” consisting of 1) symptom management web-based symptom management system using iPad at each clinic visit (20 min) and 2) coping enhancement counseling (30 min). The “Enhanced Supportive Care” will be provided by trained nurses for 5 times over 3 months. Control group participants will monitor symptoms using web-based symptom management system using iPad at each clinic visits for 5 times and their symptom report will be delivered to oncologist via EMR, whereas neither evidence based symptom management education (using iPad and booklet) nor coping enhancement counseling by research nurses will be provided.

Participants could withdraw from the study upon request, and those who transfer to different hospitals, whose condition is deteriorated for study participation will be withdrawn from the study. Upon patients’ withdrawal, caregiver will withdraw as well.

Double data entry will ensure the integrity of the data. Collected information will be coded and stored for security, and the data will be monitored every 6 months.

### Outcome measures

**Fig. 3:**
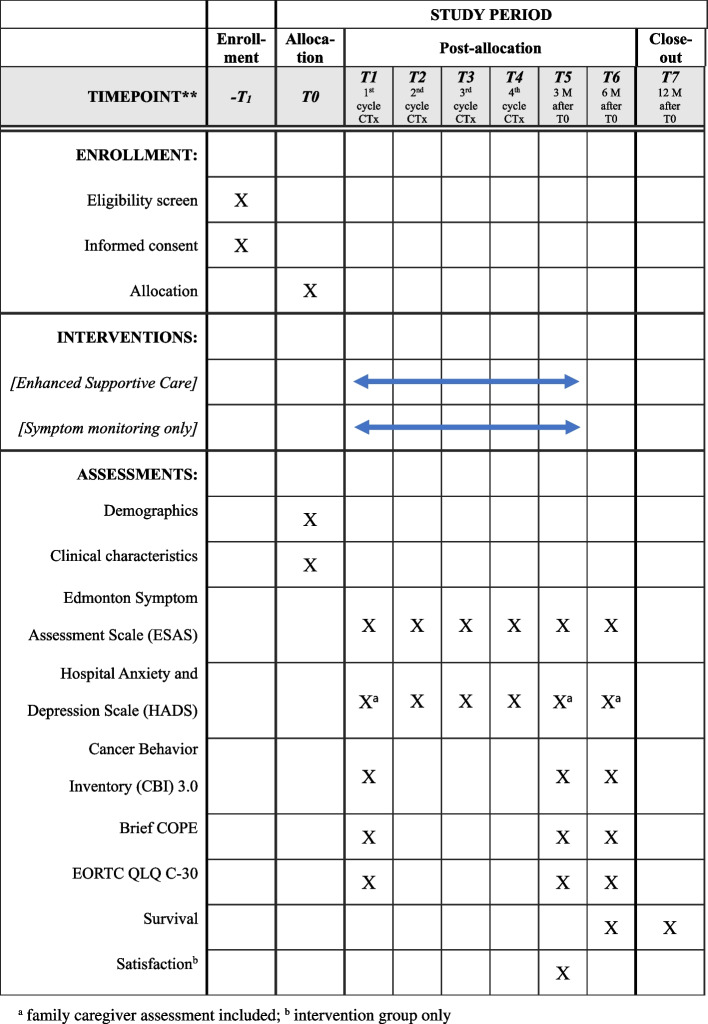
Schedule of enrollment, interventions, and assessments

The data will be collected by research nurses at seven time points: before starting the first line palliative chemotherapy (T1; baseline), on the first day of each chemotherapy from cycle 2 to 4 (T2 to T4), at 3 months (T5), 6 months (T6), and 12 months (T7) from baseline. Figure 3 outlines the data collection for the current study. Primary outcomes are symptom, coping and quality of life at 3 months. Secondary outcomes are symptom, coping, and quality of life at 6 months, depression and self-efficacy for coping with cancer at 3 and 6 months, symptom and depression change from baseline to 3 months, and survival at 6 and 12 months among patients, and depression among caregivers at 3 and 6 months.

Edmonton Symptom Assessment Scale (ESAS) [44] + 4 core symptoms recommended to be evaluated in clinical trials (constipation, diarrhea, peripheral neuropathy) [45] as well as vomiting (expert opinion) (a total of 15 items (14 symptoms and 1 well-being item)) will measure symptom. Hospital Anxiety and Depression Scale (HADS) [46] will be used to measure depression of patients and family caregivers. Self-efficacy for coping with cancer will be evaluated using the Cancer Behavior Inventory (CBI) 3.0 Korean version [47]. Brief COPE [48] and EORTC QLQ C-30 [49] will be used to evaluate coping and QoL of patients. Data will be collected at baseline (before the start of first-line palliative chemotherapy), at 3 months, at 6 months, and at 12 months. Symptoms and depression during chemotherapy cycles (1^st^ to 4^th^ CTx) will be monitored (Fig. 3).

### Endpoints

The primary endpoints are symptoms, coping, and QoL at 3 months. Secondary endpoints are symptoms, coping, and QoL at 6 months, depression and self-efficacy for coping with cancer at 3 and 6 months, symptom and depression change from baseline to 3 months, survival at 6 and 12 months among patients, and depression among caregivers at 3 and 6 months. Any withdrawal from adverse events related to the intervention will be recorded and reported.

### Sample size determination

Advanced cancer patients (*n*=360) and their family caregivers (*n*=360) will participate in the study. Sample size was estimated based on two-sided tests with α=.05, power=.80 (=.93 x .93 x .93) considering three primary endpoints [50], and medium effect size (beta >.30) for supportive care interventions for symptoms [51], coping (.50~.55) [46], and QoL (.50) [52]. The estimated sample size for t-tests was 192, that for correlation analysis was 126, and that for multiple regression with 10 control variables was 160.

Considering structural equation modeling to analyze relationships among the studied variables, a sample size of > 300 is recommended [53]. Considering longitudinal data collection and a dropout rate of 20%, a total of 360 advanced cancer patients and 360 family caregivers will be enrolled in the study.

### Statistical analyses

Per-protocol analysis will be conducted for primary endpoints using data from completers at 3 months. Intention-to-treat analysis will be conducted using the imputed full analysis set as sensitivity analysis. Missing data from those who provided data other than baseline will be imputed using the last observation carried forward (LOCF) method for symptom and depression scores. SPSS Statistics (version 26, IBM Corp., Armonk, NY, USA), SAS (version 9.4, SAS Inc., Cary, NC, USA), R (version 4.0.2, Institute for Statistics and Mathematics, Vienna, Austria, www.R-project.org), and Mplus (version 8.4, MPLUS, Los Angeles, CA, USA) software will be used for data analysis.

Primary hypothesis: Advanced cancer patients who receive “Enhanced Supportive Care” (symptom management + coping enhancement counseling) will demonstrate fewer symptoms and better coping and QoL at 3 months (T5) compared to the control group of advanced cancer patients who receive usual care with symptom monitoring only.

Secondary hypothesis: Advanced cancer patients who receive “Enhanced Supportive Care” (symptom management + coping enhancement counseling) will demonstrate fewer symptoms and better coping and QoL at 6 months (T6), less depression, higher self-efficacy for coping with cancer at 3 months (T5) and 6 months (T6). Changes in symptoms and depression from baseline (T1) to 3 months (T5) will be different between the intervention and control groups. The intervention group will demonstrate longer survival at 6 months (T6) and 12 months (T7) than control group. Family caregivers of advanced cancer patients who receive “Enhanced Supportive Care” will demonstrate less depression than family caregivers of patients who were assigned to the control group at 3 months (T5) and 6 months (T6).

#### Demographic and clinical characteristics

- Frequency and percent, mean and standard deviation (or median), minimum and maximum, and interquartile range
- Standardized difference for homogeneity for baseline characteristics between intervention and control groups

#### Primary endpoints

- Independent t-test to evaluate intervention effect for symptoms, coping and QoL at 3 months

#### Secondary endpoints

- Independent t-test to evaluate intervention effect for patients’ symptoms, coping and QoL at 6 months, patients’ depression and self-efficacy for coping with cancer at 3 and 6 months, and caregivers’ depression at 3 and 6 months
- Linear mixed model to evaluate intervention effect for change in symptoms and patients’ depression from baseline to 3 months
- Pearson correlation and multiple regression analysis to identify factors contributing to symptoms, depression, coping, self-efficacy for coping with cancer and QoL
- Kaplan‒Meier curve and log-rank test to evaluate intervention effect on survival at 6 and 12 months
- Cox proportional hazard regression to identify factors contributing to survival
- Pearson correlation analysis, mediation and moderation analysis, and structural equation modeling to understand relationships among symptoms, depression, coping, self-efficacy for coping with cancer, and QoL

## Discussion

This study will evaluate the efficacy of early palliative care comprising symptom management and coping enhancement counseling provided by nurses along with standard cancer treatment in improving the symptom experience, coping and quality of life of advanced cancer patients.

The study has several strengths. Early primary palliative care intervention will be provided by nurses to patients scheduled for first-line chemotherapy after an advanced cancer diagnosis following recommendations by the National Comprehensive Cancer Network (NCCN) and the American Society of Clinical Oncology (ASCO) [27, 30]. Early palliative care is beneficial for cancer patients in relieving symptoms and improving QoL and survival, and more than 80% of cancer patients and caregivers were positive about receiving early palliative care [54]. Early primary palliative care could be delivered by nurses because patients spend the most time with nurses compared to other healthcare professionals [55]. Nurses provide holistic care and play a critical role linking healthcare teams, patients and families in palliative care [56].

“Enhanced Supportive Care” addresses both physical and psychological management by monitoring symptoms and educating patients about self-management of symptoms from the beginning of anticancer treatment and providing counseling to enhance coping with cancer. Symptom management in “Enhanced Supportive Care” is need-based personalized symptom management education reflecting an individual’s experiences. Previous studies reported that symptom monitoring along with usual care promoted QoL and survival compared to usual care in patients with advanced cancer [57, 58]. According to a recent systematic review, patients who received education about symptom management have improved symptoms and QoL [59]. Coping enhancement counseling in “Enhanced Supportive Care” will utilize patients’ own coping resources based on principals of social cognitive theory and incorporate new perspectives in coping with cancer by introducing ACT. Promoting effective coping strategies in patients with advanced cancer during palliative care was associated with better mood and QoL [60]. Participants in the current study will be able to learn and practice symptom management and coping with cancer effectively, which may eventually improve QoL.

This study will provide data on whether addressing symptom management and coping enhancement counseling by nurses can improve symptom experience, coping and QoL among advanced cancer patients.

### Trial status

For the current RCT, participant recruitment began on 22 March 2021 and data collection will continue until 31 May 2024.

## Data Availability

The datasets generated during and/or analyzed during the current study will be available from the corresponding author on reasonable request.

## Abbreviations

ACT: Acceptance and Commitment Therapy
CBI: Cancer Behavior Inventory
EMR: Electronic Medical Record
ESAS: Edmonton Symptom Assessment Scale
CTx: Chemotherapy
EORTC QLQ- C30: European Organization for Research and Treatment of Cancer Quality of Life Questionnaire-C30
HADS: Hospital Anxiety and Depression Scale
LOCF: Last Observation Carried Forward
NHS: National Health Service
PI: Principal Investigator
RCT: Randomized Controlled Trial
WHO: World Health Organization
